# Variable and Conserved Regions of Secondary Structure in the β-Trefoil Fold: Structure Versus Function

**DOI:** 10.3389/fmolb.2022.889943

**Published:** 2022-04-19

**Authors:** Michael Blaber

**Affiliations:** Department of Biomedical Sciences, College of Medicine, Florida State University, Tallahassee, FL, United States

**Keywords:** protein symmetry, *de novo* design, hydrophobic patterning, ligand, folding nucleus

## Abstract

β-trefoil proteins exhibit an approximate C_3_ rotational symmetry. An analysis of the secondary structure for members of this diverse superfamily of proteins indicates that it is comprised of remarkably conserved β-strands and highly-divergent turn regions. A fundamental “minimal” architecture can be identified that is devoid of heterogenous and extended turn regions, and is conserved among all family members. Conversely, the different functional families of β-trefoils can potentially be identified by their unique turn patterns (or turn “signature”). Such analyses provide clues as to the evolution of the β-trefoil family, suggesting a folding/stability role for the β-strands and a functional role for turn regions. This viewpoint can also guide *de novo* protein design of β-trefoil proteins having novel functionality.

## Introduction

**FIGURE 1:**
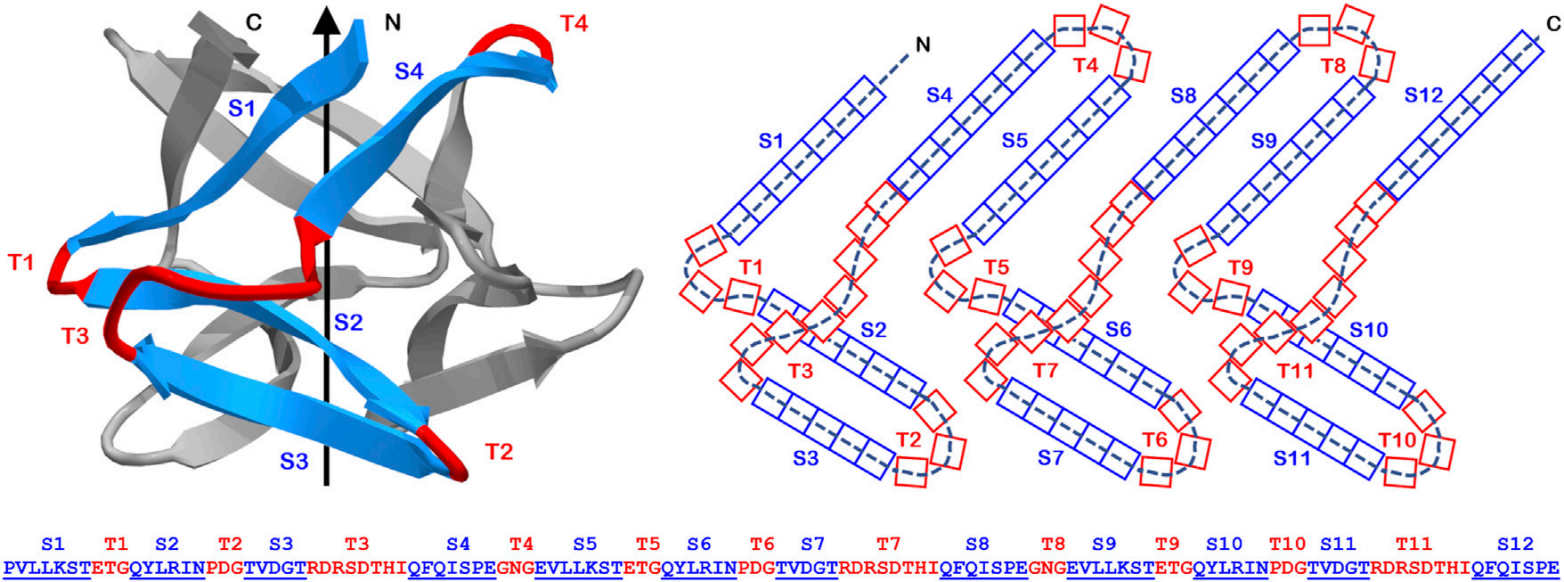
The primary, secondary, and tertiary structure of the Symfoil (“Symfoil-4T”) reference β-trefoil protein. Upper panel: A “ribbon” diagram of the Symfoil protein (RCSB 3O4B). The colored region (blue: β-strand; red: turn) identifies the first of three repeating “trefoil” motifs in the structure (the other two colored in gray). Middle panel: A two-dimensional representation of the overall β-trefoil architecture and indicating the strand and turn numbering and the number of residues in each type of secondary structure (referencing Symfoil). Lower panel: the primary structure of the Symfoil protein indicating the secondary structure positions (β-strands underlined and indicated by “S”, and turns indicated by “T”).

The β-trefoil is a common protein architecture, with 10 different superfamilies, and constituting approximately 1% of the proteome (Andreeva et al., 2013) (Table 1). A notable feature of the β-trefoil is a discernable C_3_ rotational symmetry where the repeating “trefoil” motif is approximately 40–50 amino acids in length and contains four anti-parallel β-strands connected by turn/loop regions (Sweet et al., 1974; McLachlan, 1979; Murzin et al., 1992) (Figure 1). β-trefoil proteins encompass diverse ligand-type functionalities, including toxins, protease inhibitors, cytokines, growth factors, agglutinins, lectins, and other types of ligands [SCOP database (Andreeva et al., 2019)], although no known enzymatic functionality. These ligand functionalities are associated with specific turn/loop regions that may define certain β-trefoil families (Blow et al., 1974; Veerapandian et al., 1992; Notenboom et al., 2002; Bovi et al., 2012; Blaber, 2020).

**TABLE 1:** β-trefoil superfamily and structures utilized in characterization of secondary structure heterogeneity. The overlay statistics with Symfoil-4T (RCSB 3O4B) are also provided.

Superfamily	Family	Domain	RCSB	Res. (Å)	#Cα Ovl	Ovl rmsd (Å)
Ricin B-like lectin	Ricin B-like	β-zylanase	1XYF	1.90	93	1.13
		β-galactoside-specific lectin 1	1SZ6	2.05	96	1.36
		Hemolytic lectin CEL-III	1VCL	1.70	94	1.33
		29-kDa galactose-binding lectin	2ZQO	1.80	86	1.31
		Main hemagglutinin component type C	3AH2	1.70	102	1.24
		Agglutinin	5D61	1.60	98	1.01
		Endo-1,4-β-xylanase A	1KNL	1.20	90	1.14
		Cytolethal distending toxin	1SR4	2.00	104	1.28
		Abrin-A	1ABR	2.14	95	1.22
	Cysteine rich domain	Cysteine rich domain	1FWV	1.90	88	1.19
	GlcNAc-alpha-1,4-Gal-releasing endo-β-galactosidase	GlcNAc-alpha-1,4-Gal-releasing endo-β-galactosidase	1UPS	1.82	104	1.16
	HylA β-trefoil domain-like	HylA β-trefoil domain-like	1XEZ	2.30	88	1.55
	Kunitz (STI) inhibitors	Chymotrypsin inhibitor 3	1EYL	1.90	79	1.41
		Trypsin inhibitor A	1AVW	1.75	75	1.37
		Alpha-amylase/subtilisin inhibitor	3BX1	1.85	80	1.34
		Kunitz-type serine proteinase inhibitor DrTI	1R8N	1.75	78	1.42
		Albumin-1	1WBA	1.80	74	1.34
	Clostridium neurotoxins, C-terminal domain	Botulinum neurotoxin type B	1EPW	1.90	85	1.41
		Botulinum neurotoxin type A	5MK6	1.45	79	1.19
		Tetanus toxin	1A8D	1.57	80	1.25
	Clitocypin-like	Clitocypin-5	3H6S	2.22	87	1.18
		Clitocypin-2	3H6R	1.95	89	1.26
Cytokine	Fibroblast growth factors	FGF-1	1RG8	1.10	115	1.06
		FGF-2	1BFG	1.60	113	0.98
		FGF-4	1IJT	1.80	115	1.23
		FGF-8	2FDB	2.28	110	1.23
		FGF-9	1IHK	2.20	113	1.19
		FGF-12	1Q1U	1.70	113	1.39
		FGF-19	1PWA	1.30	93	1.18
	Interleukin-1 (IL-1)	Interleukin-1 β	5R7W	1.27	95	1.34
		Interleukin-18	3WO2	2.33	89	1.33
		Interleukin-36 receptor agonist protein	1MD6	1.60	81	1.30
Actin-crosslinking proteins	Fascin	Fascin-1	3LLP	1.80	104	1.24
DNA-binding protein LAG-1 (CSL)	DNA-binding protein LAG-1 (CSL)	Lin-12 and Glp-1 phenotype	3BRD	2.21	83	1.04
AbfB domain	AbfB domain	Alpha-L-arabinofuranosidase B	1WD3	1.75	96	1.22
Agglutinin	Agglutinin	Agglutinin	1JLY	2.20	98	1.38
MIR domain	MIR domain	Inositol 1,4,5-trisphosphate receptor type 1	1N4K	2.20	101	1.12
		Uncharacterized protein (*C. elegans*)	1T9F	2.00	105	0.90
30 K Lipoprotein C-terminal domain-like	30 K Lipoprotein C-terminal domain-like	30 K protein 2	4EFP	1.33	107	1.12
		Low molecular mass 30 kDa lipoprotein 19G1	4IY9	2.10	107	1.10
		30 K lipoprotein	4PC4	1.80	104	1.10
Proteinase inhibitor 1-like	Proteinase inhibitor 1-like	Serine protease inhibitor 1	3VWC	1.50	95	1.22
*de novo* Symmetric	*de novo* Symmetric	Symfoil (Symfoil-4T variant)	3O4B	1.80	126 (Ref)	N/A (Ref)
		Threefoil	3PG0	1.62	105	1.00
		Mitsuba-1	5XG5	1.54	103	1.03

Symmetry in a subset of common protein folds has been evident from the earliest days of protein structure determination, and has stimulated hypotheses of gene duplication and fusion in their evolutionary emergence from simpler peptide motifs (Eck and Dayhoff, 1966; Ohno, 1970; McLachlan, 1972). Alternative hypotheses for such evolution of the β-trefoil have been proposed, including “emergent architecture” and “conserved architecture” models, where the simple peptide motif comprises two anti-parallel β-hairpins known as a “trefoil” (Mukhopadhyay, 2000; Ponting and Russell, 2000; Blaber and Lee, 2012; Balaji, 2015). In the emergent architecture model the structural complexity increases with each gene duplication and fusion event, such that the overall β-trefoil architecture only emerges upon a final triplet repeat of the trefoil motif. In the conserved architecture model, the trefoil peptide has the property of oligomerizing as a trimer, thereby generating an intact β-trefoil architecture. A tandem repeat also oligomerizes as a domain-swapped trimer that generates two intact β-trefoils. A triplet repeat of the trefoil motif yields a single polypeptide that folds into β-trefoil. Experimental studies lend greater support to the conserved architecture model (Lee and Blaber, 2011; Lee et al., 2011), indicating that an appropriate trefoil motif peptide can spontaneously oligomerize as a trimer to form an intact β-trefoil. Sequence and structure analyses suggest that extant β-trefoil proteins are unlikely to share a common ancestor, but are more likely to have evolved independently from simpler peptide motifs many times, and indeed, this may be a reoccurring and ongoing evolutionary process (Broom et al., 2012).

Current knowledge regarding symmetric protein architecture suggests that utilization of symmetry is an efficient and practical strategy for simplifying the *de novo* design problem (Hocker et al., 2004; Nikkhah et al., 2006; Yadid and Tawfik, 2007; Richter et al., 2010; Kopec and Lupas, 2013; Voet et al., 2014; Broom et al., 2015; Brunette et al., 2015; Huang et al., 2016; Terada et al., 2017; Afanasieva et al., 2019; Kimura et al., 2020). Furthermore, it may be practical to divide the design problem into two parts: 1) the initial design of a stable, foldable but functionless “scaffold”, followed by 2) specific functionalization (Bolon et al., 2002; Dwyer et al., 2004; Claren et al., 2009). In the case of the β-trefoil (and perhaps also the β-propeller architecture), this strategy appears especially appropriate for the design of proteins having novel ligand functionalities. It would therefore be extremely useful to elucidate the structural parameters that dictate stable, foldable architecture, from parameters that generate specific functionality.

In this report we examine the hypothesis that the structural determinants of stability and folding for the β-trefoil are principally the β-strand secondary structure (and that this is an essentially conserved structural feature in this superfamily), while specific functionality is provided by turn/loop regions (and that this is a divergent, and unique feature, among functionally-distinct β-trefoil proteins). The analysis suggests an efficient *de novo* protein design pathway that leverages symmetric principles of protein architecture.

## Materials and Methods

### Selection of Reference β-Trefoil Structure

**FIGURE 2:**
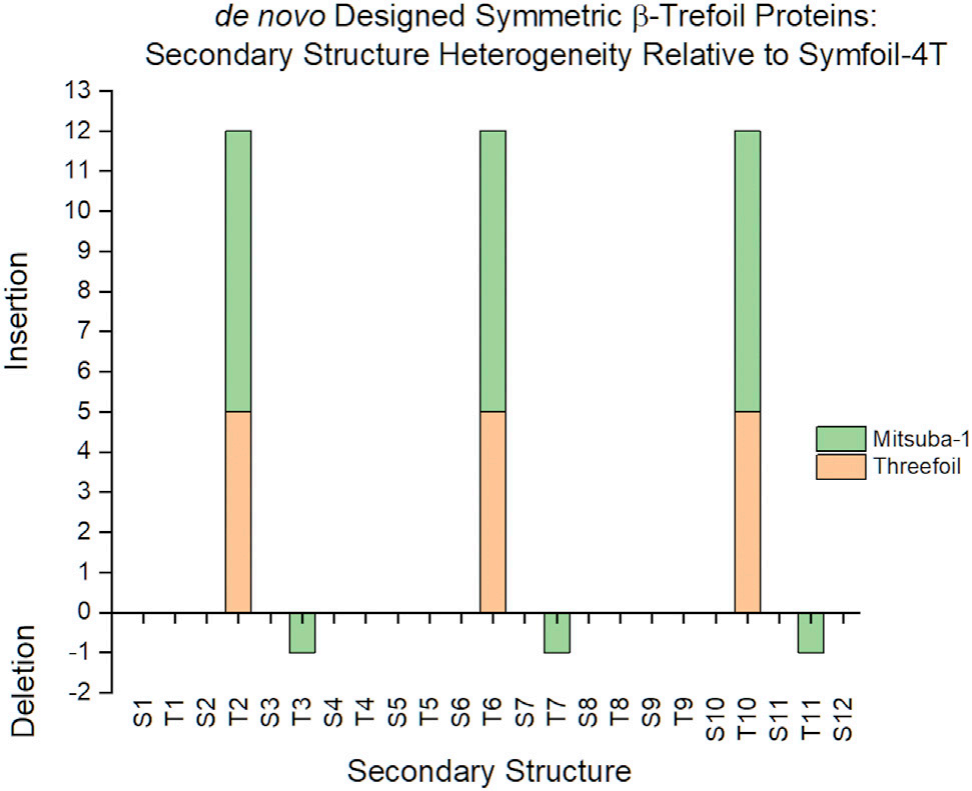
A comparison of secondary structure insertions/deletions for three symmetric designed β-trefoil proteins. The Symfoil, Threefoil, and Mitsuba-1 proteins are three independently *de novo* designed, purely-symmetric β-trefoil proteins. The most compact of these three is Symfoil, primarily due to ligand-binding turns T2, T6, and T10, engineered into both Threefoil and Misuba-1.

The identification of insertions or deletions of secondary structure within a protein architecture depends upon the reference protein used for such comparison. The reference protein should ideally comprise the essential structural architecture, with no extraneous insertions or deletions beyond the basic folding and stability requirements. In the case of the β-trefoil, where extant naturally evolved proteins exhibit varying degree of C_3_ rotational symmetry, the reference protein would ideally constitute a purely-symmetric architecture so that any asymmetric features in an evaluated protein can readily be identified. There are several *de novo* designed β-trefoil proteins having an exact threefold symmetric primary structure; including Threefoil (Broom et al., 2015), Mitsuba-1 (Terada et al., 2017), Phifoil (Longo et al., 2014) and the Symfoil family of proteins (Lee and Blaber, 2011; Lee et al., 2011). Threefoil was designed to have carbohydrate binding function and contains specific turn/loop secondary structure for this purpose. Similarly, Mitsuba-1 was designed to have a galactose binding site afforded by specific surface turn/loop secondary structure. In contrast, Symfoil was designed exclusively from the standpoint of optimized folding kinetics and thermodynamics, and is notably devoid of any specific functionality. Symfoil (using the Symfoil-4T variant) as a reference structure identifies five residue insertions within turns T2, T6 and T10 in Threefoil, and seven residue insertions of the same turns in Mitsuba-1 (Figure 2). Thus, the Symfoil protein was considered as the most appropriate reference protein with which to quantify secondary structure heterogeneity among β-trefoil proteins.

### Representative β-Trefoil Proteins

The RCSB structural databank (www.rcsb.org) was queried for β-trefoil proteins solved to better than 2.5 Å resolution. A total of 45 proteins were identified, representing 10 superfamilies, 17 families, and 45 domains, and with an average resolution of 1.81 ± 0.31 Å (Table 1). Only the *de novo* designed β-trefoil proteins exhibit an exact threefold rotational symmetry; all naturally-evolved β-trefoil proteins exhibit varying degrees of primary, secondary and tertiary structure symmetry.

### Structural Overlay

Structural overlays of individual β-trefoil proteins onto the Symfoil protein coordinates (using the Symfoil-4T variant, RCSB 3O4B) were performed using the Swiss PDB Viewer software (Guex and Peitsch, 1997) and selecting for Cα atoms. An iterative fitting process was used to optimize the overlay. The number of matching Cα atoms was noted, as well as the rmsd for the fit (Table 1). This overlay was then examined for insertions or deletions in specific secondary structure elements as defined in the Symfoil structure (Figure 1). The percent of Cα matches per secondary structure element was also determined.

### Sequence Logo Plots

Sequence logo plots are a graphical representation of an amino acid (or nucleic acid) multiple sequence alignment (Schneider and Stephens, 1990; Crooks et al., 2004). Each logo consists of stacks of symbols, one stack for each position in the sequence. The height of symbols within a stack indicates the relative frequency of each amino at that position. A sequence logo plot was generated for β-strands S1, S5, and S9 as a group; similarly, S2, S6, and S10 as a group; S3, S7, and S11 as a group; and S4, S8, and S12 as a group (i.e., all sets of C_3_ symmetry related strands, *n* = 126), for all representative β-trefoil proteins in Table 1 and using structural overlays as described above. Image generation utilized the web logo server at https://weblogo.berkeley.edu/ with colors based on chemical properties: polar amino acids (G,S,T,Y,C,Q,N) are green, basic (K,R,H) blue, acidic (D,E) red and hydrophobic (A,V,L,I,P,W,F,M) amino acids are black.

## Results

### Secondary Structure Length and Conformational Heterogeneity

**FIGURE 3:**
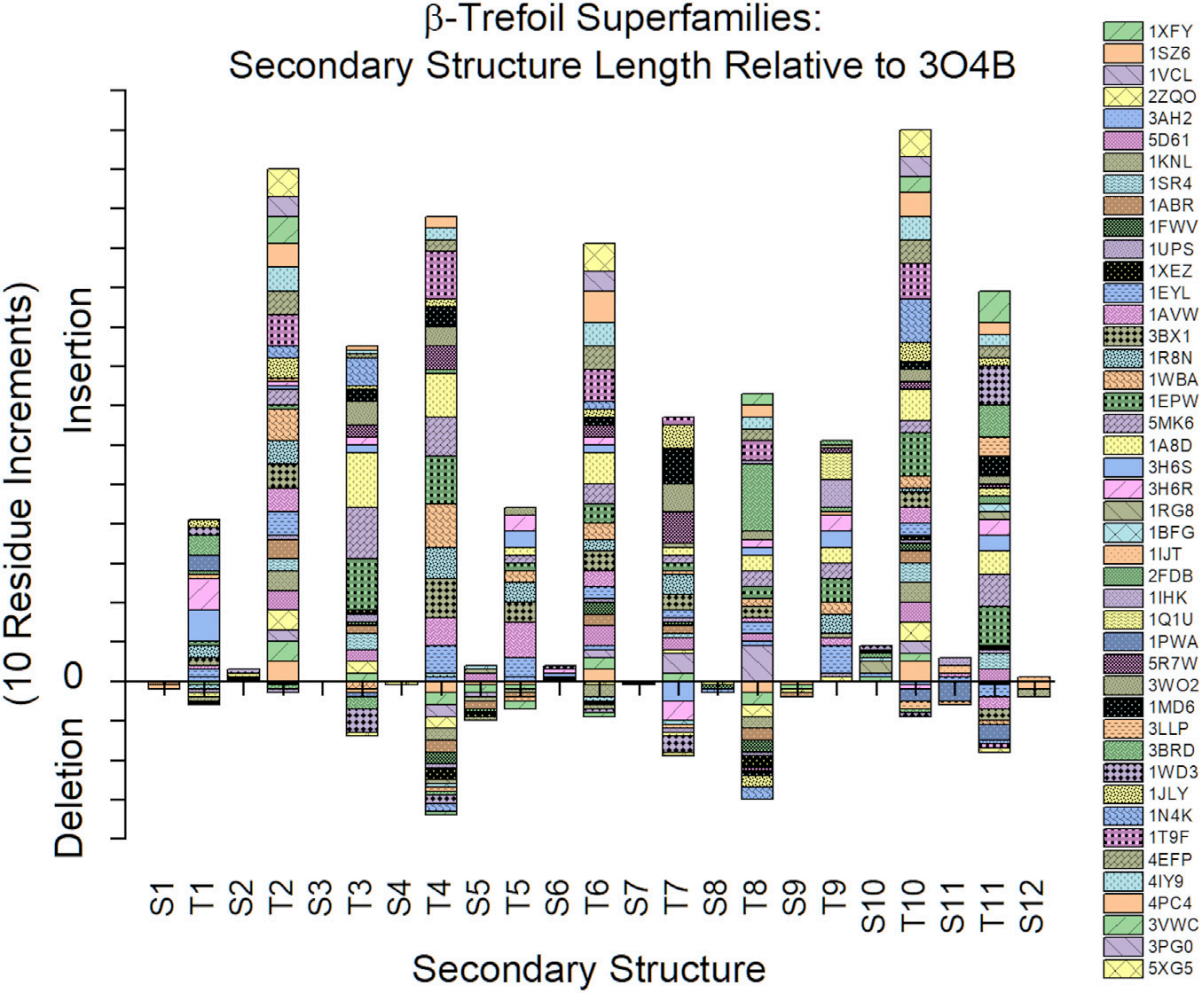
Relative insertions or deletions in secondary structure elements among the β-trefoil superfamily of proteins. The reference protein is the Symfoil protein—a *de novo* designed, purely-symmetric, minimalist, and functionless β-trefoil protein (see text).

An analysis of the secondary structure length heterogeneity for the β-trefoil superfamily of proteins, compared to the Symfoil reference, shows that the heterogeneity is localized almost exclusively to turn secondary structure; indeed, all β-strands show a remarkable absence of relative insertion or deletion (i.e., all β-strands show a marked conservation of length (Figure 3). Furthermore, the heterogeneity in the turn regions principally involves insertions, as opposed to deletions, compared to the Symfoil reference protein. However, there are two notable exceptions to this general rule at turns T4 and T8, where some β-trefoils have limited deletions of up to three amino acids.

**FIGURE 4:**
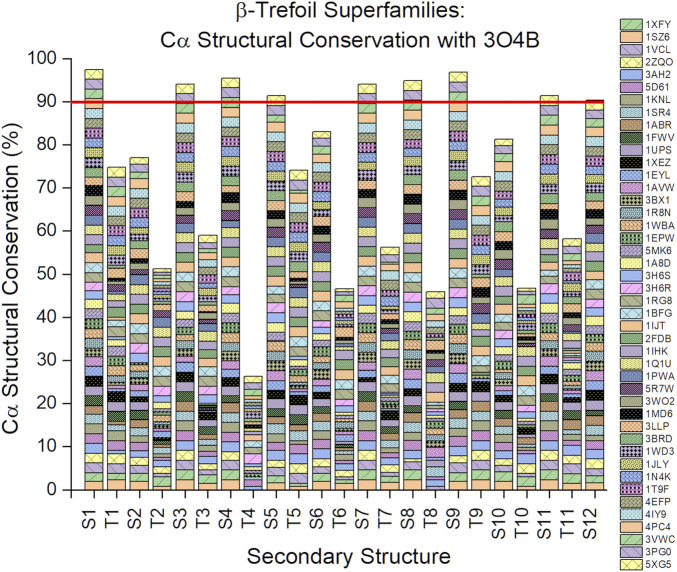
Cα structural conservation (<1.5 Å rmsd) within secondary structure elements for the β-trefoil family of proteins. The reference protein is the Symfoil protein (RCSB 3O4B)—a *de novo* designed, purely-symmetric, minimalist, and functionless β-trefoil protein (see Figure 1).

An analysis of the Cα structural conservation for regions of secondary structure in β-trefoil proteins, compared to the Symfoil-4T reference, shows that not only do β-strand regions show highly-conserved lengths, but that their overall conformation as β-strands is also highly-conserved (Figure 4). It can be seen that for the entire superfamily of β-trefoils a >90% structural conservation (i.e., <1.5 Å rmsd) is present with the symmetry-related sets of β-strands S1/S5/S9, S3/S7/S11, and S4/S8/S12. The S2/S6/S10 set exhibits 76–84% Cα structural conservation. Among turn secondary structure, turns T4 and T8 (which are symmetry-related) exhibit the least Cα structural conservation.

**FIGURE 5:**
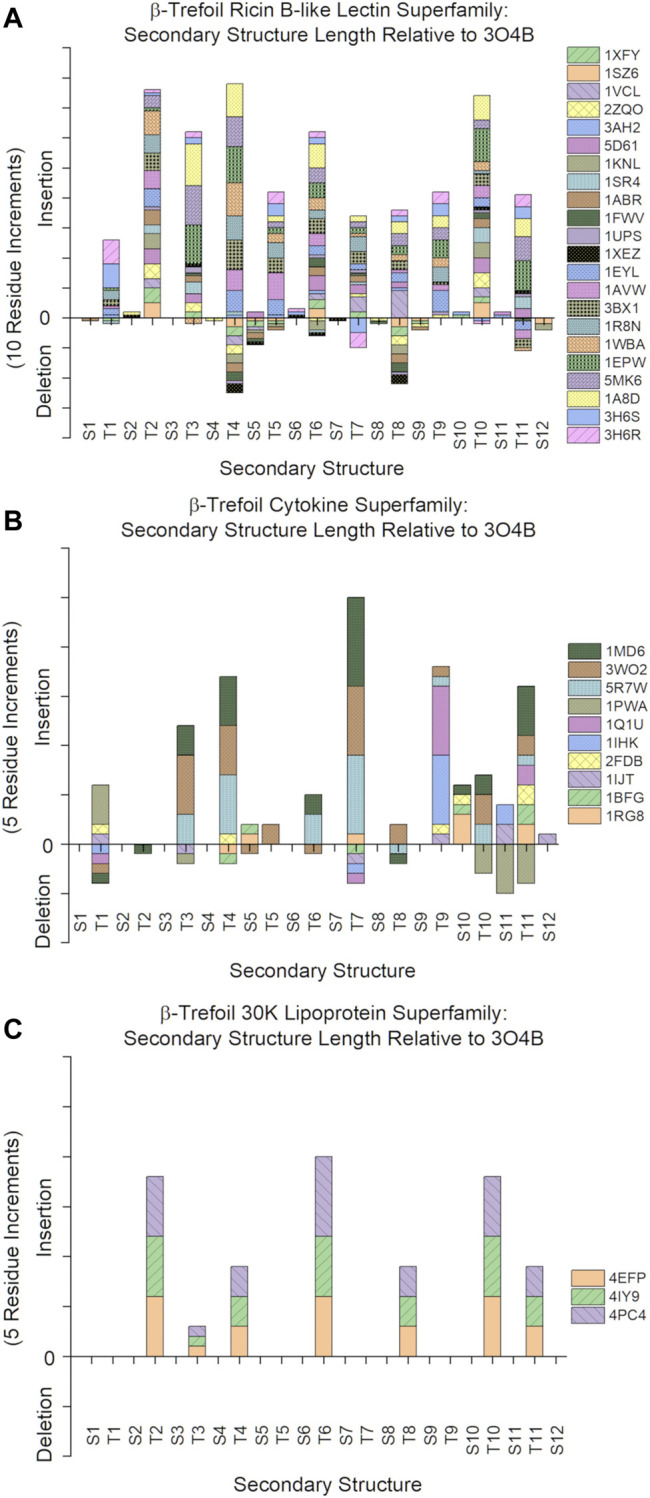
Relative secondary structure insertions or deletions of individual β-trefoil superfamilies. **(A)**: Ricin B-like lectin superfamily (*n* = 22). **(B)**: Cytokine superfamily (*n* = 10). **(C)**: 30 K Lipoprotein superfamily (*n* = 3).

The Ricin B-like, Cytokine, and 30 K Lipoprotein superfamilies have the greatest number of members, with 22, 10, and 3 members, respectively (Table 1). The secondary structure length heterogeneity for these individual families is shown in Figure 5. This graph suggests that the general turn heterogeneity observed in the overall superfamily graph (Figure 3) is a composite of patterns of turn heterogeneity unique to the individual superfamilies or families. Thus, the Ricin B-like lectin superfamily exhibits the greatest turn heterogeneity (i.e., extensions) at T2, T3, T4, T6, and T10; while the Cytokine superfamily exhibits turn extensions principally at T3, T4, T7, T9, and T11; and the 30 K Lipoprotein superfamily exhibits turn extensions principally at T2, T6, and T10. Thus, each different superfamily exhibits characteristically different turn heterogeneity (i.e., extensions).

### Sequence Logo Plots

**FIGURE 6:**
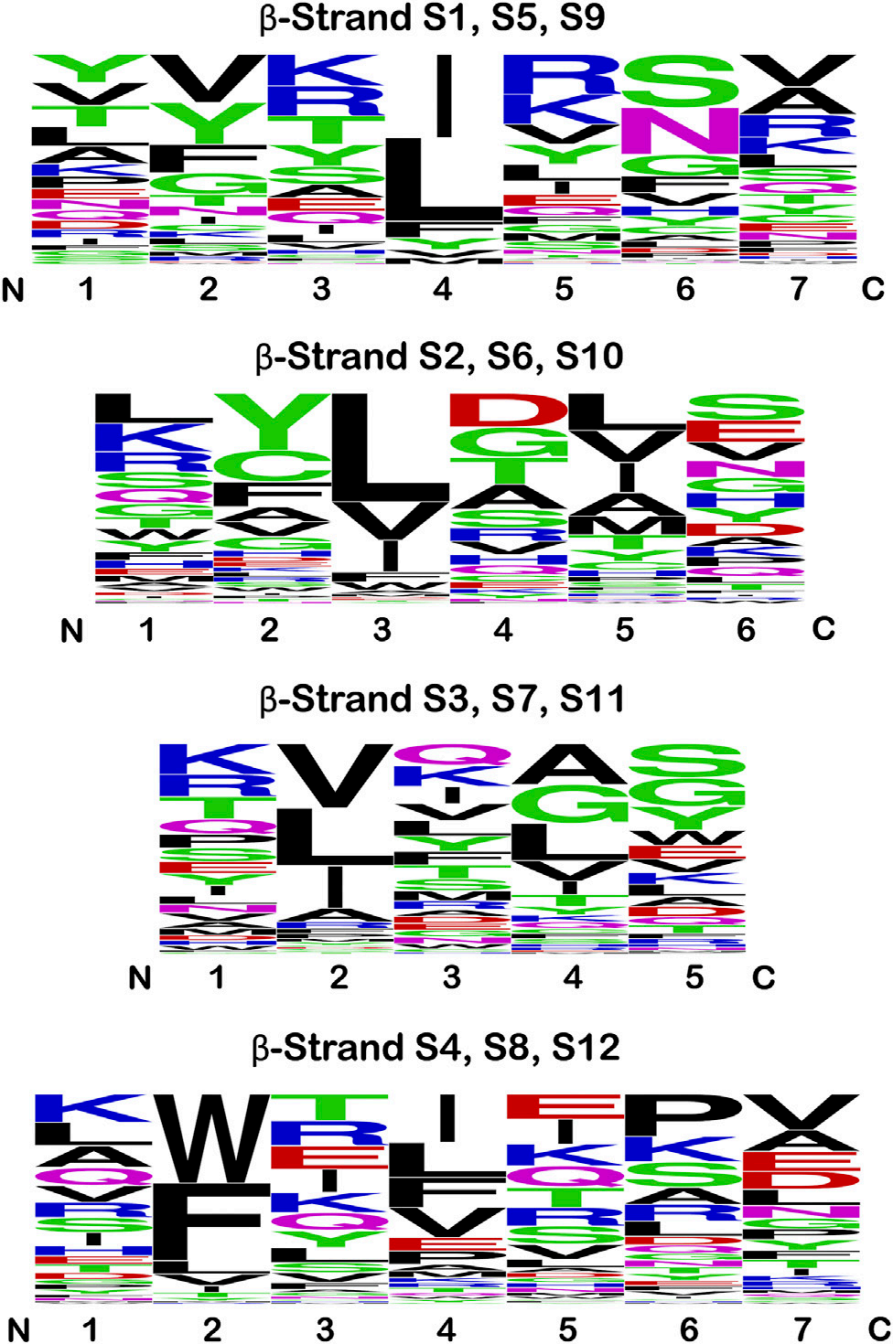
Sequence logo plots for the β-strand secondary structure in the β-trefoil superfamily. Equivalent β-strands are grouped by the C_3_ rotational symmetry of the β-trefoil for all members of the superfamily in Table 1. Thus, strands S2, S6, and S10 are grouped together in this analysis, and similarly for the other symmetry-related β-strands (therefore, *n* = 126 at each position). The single letter amino acid code is utilized, and the height indicates the relative prevalence of a particular amino acid at each position. The amino acids are colored according to chemical properties (see text); however, hydrophobic is indicated by black.

The sequence logo plots for the β-strand secondary structure exhibit characteristic patterns of hydrophobic residues (Figure 6). In β-strands S1/S5/S9 position #4 is principally hydrophobic: Ile and Leu account for 80% of all amino acids at this position, with the other residues being Phe, Tyr, Val and Met. There is some indication of hydrophobic preference at position #2, with Val and Phe accounting for approximately 40% of positions (and if Y is considered hydrophobic, then ∼50% of residues at position #2 are hydrophobic). In β-strands S2/S6/S10 positions #3 and #5 show a clear hydrophobic preference. Leu accounts for ∼50% of residues at position #3, with the majority of other residues being either Val, Ile, Phe or Trp. At position #5 Leu, Val, Ile, Ala and Met account for ∼66% of residues. In β-strands S3/S7/S11 Val, Leu and Ile account for ∼75% of residues at position #2. Ala, Leu, Val and Ile account for ∼50% of residues at position #4, with Gly another major residue at this position. In β-strands S4/S8/S12 there is a remarkable ∼70% preference of aromatic residues W or F at position #2 (with Leu, Val and Ile comprising the majority of the remainder). Hydrophobic residues are also preferred at position #4, with Ile, Leu, Phe, and Val comprising ∼60% of residues. Thus, in all β-strands there is a hydrophobic (P)/hydrophilic (H) pattern of H-P-H-P-H. Binary patterning of hydrophobic/hydrophilic amino acids is a key determinant of protein secondary structure, with an alternating hydrophobic/hydrophilic pattern favoring the formation of amphipathic β-strand secondary structure (West and Hecht, 1995; Xiong et al., 1995). These hydrophobic residues within the H-P-H-P-H patterning of the β-trefoil β-strands contribute to a highly-cooperative core packing group in the β-trefoil structure (Blaber, 2021).

## Discussion

### Is Symfoil-4T a “Minimal” β-Trefoil?

Among the *de novo* designed symmetric β-trefoil proteins Symfoil is the most compact, principally due to the absence of specific functional surface turns/loops. Analyses of structural variations (i.e., insertions or deletions) of other β-trefoil proteins indicate that the vast majority of structural heterogeneity is associated with insertions in surface turn/loop regions in comparison to Symfoil. However, there is evidence of some β-trefoil proteins having relative truncations in the T4 and T8 regions (Figures 3, 5A). Specifically, 1FWV, 1ABR, 1KNL, 2ZQO, 1SZ6, 1XYF, and 1XEZ (all members of the Ricin B-like lectin superfamily, Table 1) have three amino acid deletions in both the T4 and T8 regions. These deletions effectively eliminate the hydrophobic residue at the #2 position in the S5 and S9 β-strands (which participate in the cooperative central core); thus, these truncations of the T4 and T8 turns may result in a less stable, or less cooperatively-folding, protein. The Symfoil protein therefore represents a “minimal” or “essential” β-trefoil architecture—one that is highly-conserved in the family of β-trefoil proteins—and is therefore a useful reference structure by which to characterize secondary structure heterogeneity in β-trefoil proteins.

### Is There a Segregation of β-Strand and Turn Secondary Structure as Regards Protein Structure and Function?

The highly-conserved β-strands, and highly-divergent turn/loop regions, when comparing members of the β-trefoil superfamily, strongly suggests that functionality has its principle basis in turn/loop structure. For example, the specific heparin-binding functionality of FGF-1 (Cytokine superfamily) has been localized principally to an extension within the T11 region (Brych et al., 2004) while interaction with FGF receptor involves the T1, T4, and T8 regions (Olsen et al., 2004). Lectin functionality in the shellfish lectin MytiLec-1 and *M. oreades* mushroom lectin is localized to regions T2, T6, and T10 (Broom et al., 2015; Terada et al., 2017). The inhibitory function of Kunitz (STI) protease inhibitors is due to active site binding of an extended T4 loop region (Song and Suh, 1998). Ricin B-like lectin interactions involve the T2/T3 and T10/T11 regions (Suzuki et al., 2009). The Pmt2-MIR domain (superfamily MIR domain) interaction with tetraethylene glycol ligand involves regions T4 and T7 (Chiapparino et al., 2020). The interaction between LAG-1 (CSL) DNA-binding protein and DNA ligand principally involves the T1 region (Friedmann et al., 2008). The interaction between Agglutinin and T-disaccharide involves the T6 and T10 region (Transue et al., 1997). The interaction between clitocypin and cathepsin V involves the T1 and T3 regions (Renko et al., 2010). This representative summary of binding interactions provides strong support for a primary assignment of functionality to specific and structurally-heterogenous turn/loop regions in β-trefoil proteins.

### Can Turn Structure Provide Evidence of Evolutionary Gene Duplication/Fusion Processes?

**FIGURE 7:**
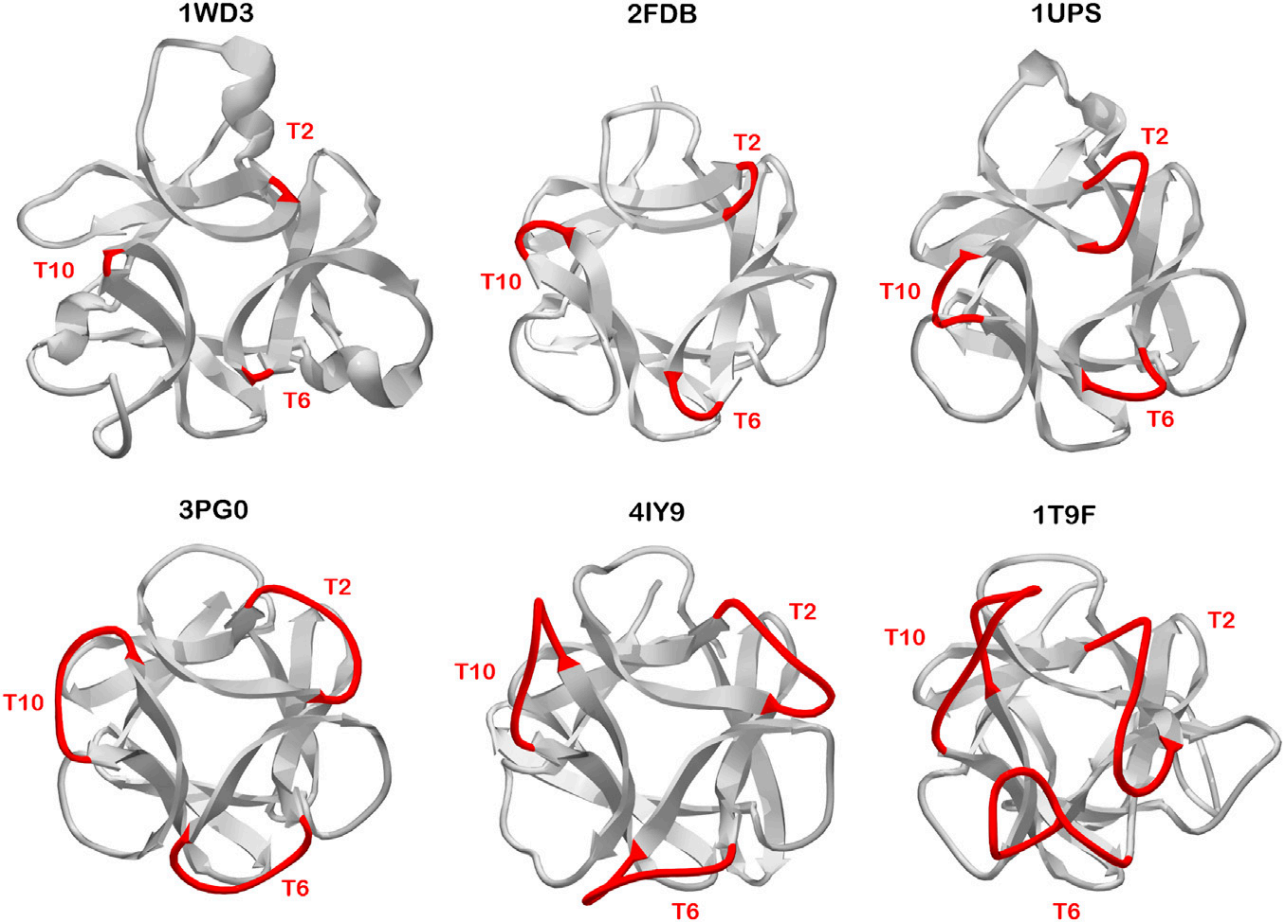
Examples of β-trefoil proteins having distinct C_3_ symmetry at the T2/T6/T10 turn region. The turn length in reference to the Symfoil protein is -1 (3PG0), 0 (2FDB), +1 (1UPS), +5 (3PG0), +6 (4IY9), and +8 (1T9F). The view is down the C_3_ axis of rotational symmetry. Such symmetric relationships in turn structure suggests divergence of this turn structure occurred prior to duplication/fusion/truncation events leading to the extant β-trefoil architecture.

Symmetric relationships among turn/loop structures in β-trefoils appears most apparent within the symmetry-related set of T2/T6/T10 turn positions. There are β-trefoil proteins having relative insertions of *n* = +1 (1UPS), *n* = +5 (3PG0), *n* = +6 (4IY9), *n* = +7 (5XG5), and *n* = +8 (1T9F) amino acids, relative to the Symfoil (i.e., 3O4B) reference structure. Additionally, similar examples exist having no relative insertions (i.e., *n* = 0; 1Q1U/1IHK/2FDB/1IJT/1BFG/1RG8) as well as *n* = −1 deletions (1WD3) (Figure 7). The most parsimonious explanation for such structural conservation of these symmetry-related turns is for duplication/fusion events to occur subsequent to trefoil motif structural evolution. This implies the likelihood of multiple independent instances of the evolution of β-trefoil proteins from simpler (i.e., trefoil-fold) motifs, and supports the evolutionary hypothesis put forth by Meiering (Broom et al., 2012) that the emergence of β-trefoil proteins is a recurring and ongoing evolutionary mechanism.

In the simplest example of duplication and fusion of individual trefoil-motifs leading ultimately to formation of a β-trefoil protein, the junction of gene fusion is the T4 turn region (Ponting and Russell, 2000; Lee and Blaber, 2011; Lee et al., 2011). Thus, the β-trefoil architecture contains two symmetry-related turns T4 and T8, with the “third” member of this symmetrically-related set defined by the adjacent (but discontinuous) N- and C- termini (see Figure 1). As with the T2/T6/T10 turns, a number of β-trefoil proteins exhibit a unique structural symmetry when comparing the T4 and T8 turns (e.g., 1FWV, 1ABR, 1KNL, 2ZQO, 1SZ6, 1XYF, 1XEZ; as described above). This implies that this turn formed prior to the duplication and fusion event that yielded the mature β-trefoil architecture. However, this results in a structural conundrum. The existence of a T4 region results from the fusion of two trefoil motifs. Two such turns (i.e., T4 and T8) would be generated by a subsequent tandem duplication of such a construct; however, this would yield a total of four sequential trefoil motifs. The apparent solution to the presence of an “extra” trefoil motif is for the latter fusion to include a truncation event affecting one trefoil motif (Jeltsch, 1999; Peisajovich et al., 2006; Longo et al., 2013).

### Turns and the Folding Nucleus

In addition to providing a potential functional role, turns also serve to connect adjacent β-strand secondary structure (forming a β-hairpin), minimizing the entropic penalty of association, and thereby influencing stability and folding (Nagi et al., 1999; Thompson and Eisenberg, 1999; Lindberg et al., 2006). The reaction coordinate of cooperative protein folding typically describes a highly-polarized transition state or folding nucleus (Abkevich et al., 1994; Went and Jackson, 2005; Faísca, 2009). Establishment of this folding nucleus is the rate limiting step in folding, and once formed, serves to rapidly condense formation of the overall native structure. An isolated 42-mer trefoil motif (i.e., “Monofoil”) derived from the Symfoil protein spontaneously oligomerizes to yield an intact β-trefoil architecture (Lee and Blaber, 2011; Lee et al., 2011); thus, a serviceable folding nucleus resides within each repeating motif in the Symfoil protein (Blaber, 2020; Parker et al., 2021). However, phi-value analysis (Fersht and Sato, 2004) indicates that the effective folding nucleus in the Symfoil protein, and the related fibroblast-growth factor-1 β-trefoil protein, while not identical, are both centrally-located and more expansive than an individual trefoil motif (Longo et al., 2014; Xia et al., 2016). This more expansive central definition includes turns T4 and T8, which are novel turn structures generated by the fusion of trefoil motif repeats. These novel turns are postulated to promote local β-hairpin interactions, thereby generating a more efficient folding nucleus compared to an isolated trefoil motif. However, destabilizing mutations targeting the folding nucleus region of Symfoil indicate that the C_3_ symmetry provides for alternative folding nuclei in other regions of the protein able to salvage foldability (Longo et al., 2013; Tenorio et al., 2020). The survey of turn region lengths in the β-trefoil superfamily indicates that the central region comprises turns having generally the shortest lengths (Figure 3). Thus, central turns may be somewhat “privileged” regions of secondary structure where considerations of efficient folding nucleus formation impact the optimal turn length and sequence design.

### Implications and Suitability of β-Trefoil Proteins for *de novo* Design

**FIGURE 8:**
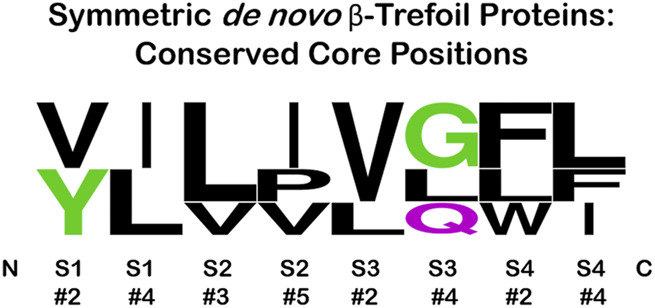
Sequence logo plot of the set of symmetric core-packing residues (see Figure 6) present in *de novo* designed symmetric β-trefoil proteins Symfoil-4T (3O4B), Phifoil (4OW4), Threefoil (3PG0), and Misuba-1 (5XG5). The positions within a single trefoil motif are shown, but these are replicated exactly for the other two trefoil motifs in each protein. Position #4 in S1, and position #3 in S2, have the highest neighbor contacts among the set of core residues (Blaber, 2021).

The secondary structure elements of the fundamental β-trefoil are limited to β-strand and reverse turn, and thus describe a comparatively simple protein architecture. Knowledge essential for the *de novo* design of β-trefoil proteins is extensive: 1) The β-strand secondary structure is the key determinant of the conserved basic architecture for this protein superfamily; 2) Conserved β-strand characteristics have been elucidated as regards length and hydrophobic patterning; and 3) The role of β-strand hydrophobic residues in cooperative core-packing interactions has been well-characterized. In this regard, it is interesting to note the different independent solutions for the set of hydrophobic core-packing residues (referencing Figure 6) utilized by the *de novo* designed symmetric β-trefoil proteins Symfoil [3O4B; generated through top-down symmetric deconstruction of FGF-1 (Lee and Blaber, 2011; Lee et al., 2011)], Phifoil [4O4W; generated by folding nucleus symmetric expansion of FGF-1 (Longo et al., 2014)], Threefoil [3PG0; generated by consensus sequence of a carbohydrate-binding ricin sequence (Broom et al., 2012)], and Mitsuba-1 [5XG5; generated by computational sequence constraint of the shellfish lectin MytiLec-1 (Terada et al., 2017)]. The sequence logo plot for this set of core-packing residues (Figure 8) suggests that, as long as the appropriate hydrophobic patterning and compatible van der Waals interactions are satisfied, a variety of alternative core-packing arrangements are permissible, thereby indicating a lowered threshold for successful design.

The general attributes of the folding nucleus for Symfoil have been identified, and the potential for redundant folding nuclei demonstrated. Evolutionary considerations indicate highly-permissive design pathways of foldability involving diverse fusion/truncation of trefoil motifs. Turn regions have been identified as the key regions of structural variability, and are the principle determinants of ligand functionality characteristic of this superfamily. As connectors of adjacent β-strand secondary structure, turn regions also influence the entropic penalty for the assembly of local β-hairpin structure, and this plays an important role in the formation of the folding nucleus.

Protein design must simultaneously solve at least three different problems: 1) protein foldability (i.e., folding kinetics requirements), 2) protein stability (i.e., thermodynamic requirements), and 3) the accommodation of specific function (with potential structural dynamics requirements). Analysis of the β-trefoil architecture suggests that it is readily amenable to a two-step design process, with the initial step focusing upon the design of a foldable, stable “scaffold” (and many avenues appear possible); subsequently followed by a second step of functional mutation. The present analysis indicates that the first step involves β-strand secondary structure and key hydrophobic patterning design (building upon current extensive knowledge in this area). The C_3_ symmetry substantially reduces the combinatorial search of appropriate primary structure solutions. The second step focuses upon turn/loop regions and their mutation to generate desired functionality (the β-trefoil architecture perhaps best suited to ligand functionality). This second step is less-well characterized and therefore open to expansive and novel opportunities. The C_3_ symmetry provides for monovalent or multivalent ligand binding opportunities. In an alternative approach, if specific loop regions are associated with unique functional properties, and the β-strands as structural elements, then diverse chimeras with novel combined structure/function attributes might be constructed using computational approaches (Ferruz et al., 2021). Overall, the β-trefoil architecture has many attractive features for *de novo* protein design, applied especially to ligand functionality. The adoption of heparin-binding functionality into a benign β-trefoil scaffold using the principles described herein has recently been demonstrated (Tenorio et al., Forthcoming 2022).

## Data Availability

The original contributions presented in the study are included in the article/Supplementary Material, further inquiries can be directed to the corresponding author.
